# Natural Products with Potential Effects on Hemorrhoids: A Review

**DOI:** 10.3390/molecules29112673

**Published:** 2024-06-05

**Authors:** Yicheng Liang, Tankun Ren, Ruyi Li, Zhonghui Yu, Yu Wang, Xin Zhang, Zonglin Qin, Jinlong Li, Jing Hu, Chuanhong Luo

**Affiliations:** 1 State Key Laboratory of Southwestern Chinese Medicine Resources, Pharmacy College, Chengdu University of Traditional Chinese Medicine, Chengdu 611137, China; hcaaqaam@163.com (Y.L.); 15181497960@163.com (T.R.); 15908167906@163.com (R.L.); wy1899098639@163.com (Y.W.); m18982414026@163.com (X.Z.); m17723861480@163.com (Z.Q.); ypl020228@163.com (J.L.); 2 School of Clinical Medicine, North Sichuan Medical College, Nanchong 637002, China; yzhzz0512@163.com

**Keywords:** hemorrhoids, hemorrhoid improvement, natural products, pharmacological mechanisms

## Abstract

Hemorrhoid disease is a common anorectal disorder affecting populations worldwide, with high prevalence, treatment difficulties, and considerable treatment costs. Compared to other treatment options, medical therapy for hemorrhoids offers minimal harm, more dignity to patients, and is more economical. Unfortunately, there are few chemical hemorrhoid medications available clinically, which makes the search for efficacious, cost-effective, and environmentally friendly new medication classes a focal point of research. In this context, searching for available natural products to improve hemorrhoids exhibits tremendous potential. These products are derived from nature, predominantly from plants, with a minor portion coming from animals, fungi, and algae. They have excellent coagulation pathway regulation, anti-inflammatory, antibacterial, and tissue regeneration activities. Therefore, we take the view that they are a class of potential hemorrhoid drugs, prevention products, and medication add-on ingredients. This article first reviews the factors contributing to the development of hemorrhoids, types, primary symptoms, and the mechanisms of natural products for hemorrhoids. Building on this foundation, we screened natural products with potential hemorrhoid improvement activity, including polyphenols and flavonoids, terpenes, polysaccharides, and other types.

## 1. Introduction

**Figure 1:**
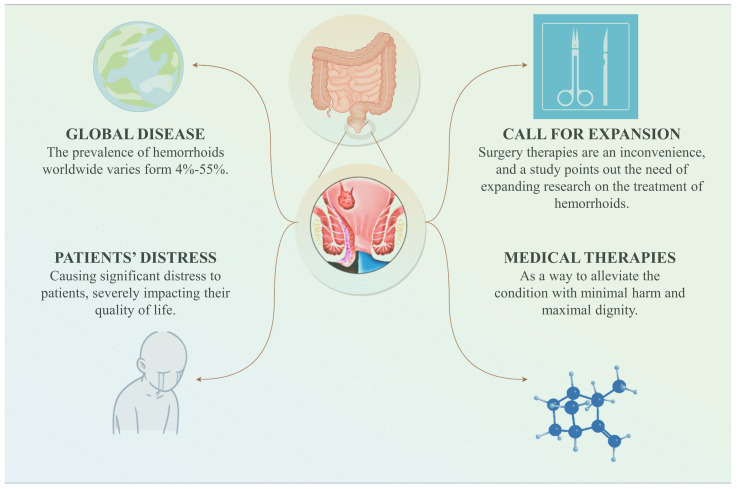
Current status of hemorrhoids.

Hemorrhoids are a type of global disease that encompasses external, internal, and mixed hemorrhoids. The prevalence of hemorrhoids worldwide varies from 4% to 55% [1]. An epidemiological survey of common anorectal diseases among urban residents in China revealed that 51.14% of adults suffered from anorectal diseases, with hemorrhoids accounting for 50.28% of these cases [2]. The complications arising from hemorrhoids often cause significant distress to patients, severely impacting their quality of life. Hemorrhoids have evidently become a major health concern that threatens human well-being. Meanwhile, a study on the economic burden of hemorrhoids in the United States highlights the need to expand research on the etiology, prevention, and treatment of hemorrhoids, considering the escalating costs of treatment and the substantial economic burden [3] (Figure 1).

Hemorrhoid disease is defined as the symptomatic enlargement and distal displacement of the normal anal cushions. The main symptoms of hemorrhoids include bleeding, inflammation, pain, infection, pruritus, ulceration, prolapse, and mucus exudation. Currently, the hemorrhoid medications with evidence of effectiveness include diosmin, troxerutin, hydroxyethylrutoside, and calcium dobesilate, which can significantly improve hemorrhoid bleeding, inflammation, and pruritus [4,5,6], etc. 

Natural therapies, which are advocated by the guidelines of the European Society of Coloproctology on hemorrhoid disease management, include bran, psyllium, and senna, to alleviate symptoms and bleeding associated with hemorrhoids [7]. Meanwhile, in the American Society of Colon and Rectal Surgeons Clinical Practice Guidelines for the Management of Hemorrhoids [8], medical therapies are highlighted as a means to alleviate the condition with minimal harm and maximal dignity, specifically mentioning the use of phlebotonics, particularly flavonoids, in the improvement of both acute and chronic hemorrhoid disease. Iranian traditional medicine, on the other hand, utilizes natural medicines like *Allium ampeloprasum*, *Phyllanthus emblica,* and *Aloe vera* in the improvement of hemorrhoids and their attendant symptoms [9]. In China, comprehension of hemorrhoid disease can be traced back approximately 2000 years to the *Classic of Mountains and Rivers* (Shan Hai Jing), where various natural medicines, including *Sanguisorba officinalis* L. (Di Yu) and dried flowers and buds of *Sophora japonica* L. (Huai Hua), are often employed as therapeutic medicines for hemorrhoids [10]. These instances collectively illustrate the potential of natural therapies in the improvement of hemorrhoids.

According to the above facts, utilizing natural medicines or natural products for hemorrhoid improvement is a viable approach, yet the clinical application of natural medicines remains limited due to low evidential support, unclear active ingredients, and other factors, leading to their role primarily as complementary and alternative therapies. Furthermore, the number of approved chemical drugs with hemorrhoid improvement effects is very limited [3]. Therefore, finding natural products that are cost-effective, efficacious, have clear pharmacological mechanisms, and are safe for hemorrhoid improvement is significant. This review aims to provide some reference bioactive components for the future development of hemorrhoid medications and prevention products. At the same time, it offers reference for improving the composition of existing hemorrhoid medications by integrating the etiology, classification, and natural product pharmacological mechanisms of hemorrhoids.

## 2. Etiology, Classification, and Mechanisms of Natural Products for Hemorrhoids

### 2.1. Etiology

Hemorrhoid disease represents a complex ailment for which the pathogenic mechanisms remain unclear [11]. The etiology of hemorrhoids is typically multifactorial. Predominant contributing factors include human physiological structure [12], dietary habits, lack of physical exercise, and defecatory behaviors [4]. These factors collectively increase intra-abdominal pressure during bowel movements, adversely affecting blood flow in the anal and rectal vessels, which may lead to swelling, bleeding, and the development of hemorrhoids [13]. Additionally, inflammatory responses [14], dysbiosis of the intestinal microbiota [15], and perianal infectious abscesses can also indirectly contribute to the onset of hemorrhoids [16]. Lastly, certain specific conditions such as sexual activity [17], pregnancy [18], and genetic factors [17] may also be associated with the development of hemorrhoids. Furthermore, in a sample of 4984 patients, it was shown that of these factors [17], defecation factors were present in 1455 patients (about 29.19%), exercise factors in 1231 patients (24.70%), sex factors in 1046 patients (20.99%), dietary factors in 923 patients (18.52%), and 329 cases (6.60%) had genetic factors. And a review reported a prevalence of between 25% and 35% in pregnant women [19].

### 2.2. Classification and Symptoms

Hemorrhoids can be classified into three types according to their location: internal, external, and mixed types. The statistics of different countries and regions are not the same; in China, internal types accounted for 52.23% of all hemorrhoids, which refer to pathological changes in the submucosal venous plexus that lead to the formation of hemorrhoidal cores, which can be graded into four degrees according to the Goligher classification system [20,21] (Table 1). The main clinical manifestations of internal types are bleeding, prolapse, and pruritus, and complications such as thrombosis, incarceration, strangulation, and defecation difficulty may also occur [22]. External types (14.04%) occur below the dentate line and are caused by damage to the anal venous plexus or persistent and recurrent inflammation, blood stasis, thrombosis, or anal tissue hyperplasia. The main symptoms of external hemorrhoids are pain and swelling due to inflammation and thrombosis, which can cause severe discomfort during defecation. Based on histopathological characteristics, external hemorrhoids can be further categorized into four types: connective tissue external hemorrhoids, varicose vein external hemorrhoids, thrombotic external hemorrhoids, and inflammatory external hemorrhoids [22]. Mixed types (21.05%) refer to the presence of both internal and external hemorrhoids, where the vascular plexuses of the internal and corresponding external hemorrhoids merge across the dentate line, forming a single entity. Mixed hemorrhoids can present with symptoms of both internal and external hemorrhoids and, in severe cases, may prolapse in the form of circumferential hemorrhoids [22,23].

### 2.3. Main Mechanisms of Natural Products’ Effects on Hemorrhoids 

#### 2.3.1. Hemostasis

Internal hemorrhoids and mixed hemorrhoids frequently manifest with bleeding symptoms [22,23]. Therefore, hemostasis plays an important role in the improvement of hemorrhoids. Hemostasis is a complex biological process that initiates with the activation of coagulation factors near the site of injury or damage, ultimately leading to the formation of a fibrin clot, which prevents bleeding at the wound site [24]. 

Upon bleeding, blood vessels are damaged, and platelets are initially activated and begin to aggregate at the site of injury. Platelets adhere to the inner wall of the damaged blood vessel through the process of platelet adhesion. Subsequently, platelets release various chemical substances, such as platelet-activating factor (PAF), which promote further aggregation of platelets, forming a platelet thrombus.

The production of coagulation factor IIa (thrombin) is central to the coagulation process. It can be activated via two pathways: intrinsic pathway and extrinsic pathway. The intrinsic pathway, which is initiated when factor XII encounters collagen fibers upon damage to the vascular endothelium, activated to factor XIIa. The primary function of factor XIIa is to activate factor XI to factor XIa, thus initiating the intrinsic coagulation pathway, which ultimately activates factor X. The extrinsic pathway, which is initiated when tissue damage releases tissue factor (factor III), which forms a 1:1 complex with factor VII. This complex can rapidly activate factor X in the presence of calcium ions and phospholipids, and it can also activate factor IX to factor IXa in the presence of calcium ions. Factor IXa, in conjunction with factor VIII, activates factor X. Both pathways converge into a common coagulation pathway, where the activated factor X (Xa), together with factor V, phospholipids, and calcium ions, forms the prothrombinase complex. This complex catalyzes the conversion of factor II (prothrombin) to factor IIa. The primary function of factor IIa is to catalyze the transformation of fibrinogen into insoluble fibrin. Fibrin forms a network that traps platelets and other blood cells, forming a stable thrombus. Additionally, the coagulation process is regulated by other mechanisms to ensure that thrombi form only when necessary and to prevent excessive thrombus formation. Anticoagulant proteins (such as antithrombin III and protein C) and the fibrinolytic system participate in this regulatory process, helping to dissolve excess thrombi and maintain normal vascular function.

Therefore, natural products can achieve hemostatic effects through the following aspects [25,26]: promoting the first and second phases of aggregation platelets, promoting the processes of the intrinsic and extrinsic coagulation pathways, and inhibiting the fibrinolytic system. Additionally, promoting the contraction of vascular smooth muscle to decrease blood flow, thereby reducing the time required for coagulation at sites of vascular damage, is also a possible way.

#### 2.3.2. Anti-Inflammation

Multiple symptoms of hemorrhoids are closely associated with inflammation [14,27,28]. Such as pain, redness and swelling, anal discomfort, and thrombosis. Inflammatory cytokines and enzymes, such as interleukin-6 (IL-6), interleukin-17 (IL-17), tumor necrosis factor-alpha (TNF-α), nitric oxide (NO), inducible nitric oxide synthase (iNOS), and matrix metalloproteinases (MMPs), show a high correlation with the pathogenesis of hemorrhoids. Additionally, some inflammatory cells are also present within hemorrhoidal tissue. Therefore, anti-inflammatory action is an important criterion for evaluating the efficacy of natural products for hemorrhoid improvement.

The inflammatory response is a defensive reaction of the body to infection, injury, or other stimuli, involving the activities of numerous signaling pathways, cells, and molecules. Among these, various signaling pathways play crucial roles [29,30,31]. Mainly including the arachidonic acid (AA) pathway, mitogen-activated protein kinase (MAPK) pathway, nuclear factor kappa B (NF-κB) pathway, and activator protein-1 (AP-1) pathway. Among them, the MAPK, NF-Κb, and AP-1 pathways are initiated by activation of Toll-like receptors (TRLs).

##### AA Pathway

AA is an important precursor to inflammatory mediators. Under the action of cyclooxygenase (COX) and lipoxygenase (LOX), it can be converted into prostaglandins (PGs) and leukotrienes (LTs). These inflammatory mediators, by binding to their respective receptors, activate downstream signaling pathways and promote the onset of inflammation. Among them, COX-2 is upregulated during inflammatory processes, catalyzing the conversion of arachidonic acid into prostaglandin E2 (PGE2), which facilitates the release of inflammatory factors and the progression of inflammatory responses, such as increased vascular permeability, pain, and fever. Additionally, malondialdehyde (MDA) can act as an agonist of COX, promoting the conversion of AA to PGs, and can also influence the synthesis pathway of LTs, leading to the occurrence of inflammation [32].

##### MAPK Pathway

MAPKs are a group of serine/threonine kinases. The MAPK pathways are involved in various inflammatory processes, such as promoting cytokine production, immune cell activation, and apoptosis. Key MAPK pathways include the extracellular signal-regulated kinase (ERK), c-Jun N-terminal kinase (JNK), and p38 pathways, etc. Inflammatory stimuli can activate these MAPK pathways, leading to the phosphorylation of downstream transcription factors such as AP-1 and NF-κB. This activation plays a crucial role in the regulation and expression of genes involved in the inflammatory response.

##### NF-κB Pathway

NF-κB is a critical transcription factor that plays a pivotal role in inflammatory responses. Under normal conditions, NF-κB is bound to the inhibitory protein IκB in an inactive state within the cytoplasm. When cells are subjected to inflammatory stimuli, IκB kinase (IKK) is activated, leading to the phosphorylation and subsequent degradation of IκB. The liberated NF-κB then translocates to the nucleus, where it binds to the promoter regions of target genes, initiating the transcription of inflammation-related genes such as the inflammatory cytokines IL-1β, IL-6, and TNF-α, and inflammatory enzymes like COX-2 and Inos.

##### AP-1 Pathway

AP-1 is a class of transcription factors comprising the Jun and Fos protein families. AP-1 is involved in the regulation of several inflammatory cytokines and enzymes, such as IL-1, IL-6, TNF-α, COX-2, and MMPs. It also collaborates with NF-κB to jointly regulate inflammatory responses. The activity of AP-1 is modulated by the MAPK pathway and other signaling pathways. MAPKs phosphorylate and activate Jun and Fos proteins, facilitating the binding of AP-1 to the promoter regions of inflammation-related genes, thereby controlling the transcription of these genes.

In summary, the mechanisms of anti-inflammatory natural products primarily involve [29] regulating inflammatory signaling pathways, acting on inflammation receptors, and controlling the biosynthesis of inflammatory mediators (pro-inflammatory and anti-inflammatory factors).

#### 2.3.3. Antibacterial

Symptoms of hemorrhoids, such as perianal abscesses and anal pruritus, are intricately linked to the proliferation of pathogenic bacteria in the perianal area. Postoperative interventions for hemorrhoids include the use of antimicrobial medicines, which are pivotal in preventing postoperative complications and fundamentally facilitating the improvement of the patient’s surgical wounds [33]. The primary pathogens in the perianal region are bacteria such as *Escherichia coli* (*E. coli*), *Klebsiella pneumoniae*, and *Staphylococcus aureus*. Furthermore, antibiotic-resistant strains of *E. coli* have been isolated from perianal abscesses [34]. Current research underscores that *E. coli* is the most frequently occurring causative agent of perianal abscesses [35].

The mechanisms of antimicrobial activity of natural products are diverse [36,37,38,39]. Some investigations into antimicrobial mechanisms have demonstrated that certain medicines exhibit antimicrobial activity by disrupting cell membranes or interacting with them. This includes capabilities such as compromising the bacterial cell envelope structure, which may lead to the formation of pores, leakage, changes in electrical charge, alterations in polarity, increased permeability, and modifications in membrane fluidity. Other antibacterial mechanisms of natural products include suppressing nucleic acid synthesis, altering functions of the cytoplasmic membrane, suppressing energy metabolism, reducing cellular adhesion and biofilm formation, inhibiting the function of porin on the cell membrane, changing membrane permeability, and attenuating pathogenicity.

Overall, natural products primarily inhibit the growth of perianal pathogenic bacteria and their toxin production through mechanisms such as promoting bacterial apoptosis, disrupting bacterial cell walls, inhibiting DNA replication and transcription, suppressing bacterial protein synthesis, altering cell membrane permeability, inhibiting bacterial energy metabolism, and reducing pathogenicity.

#### 2.3.4. Tissue Regeneration

The healing of tissue damage following hemorrhoid rupture is a complex physiological process, which can be broadly divided into four stages: hemostasis, inflammation, proliferation, and remodeling. Multiple signaling pathways and mediators are involved in this tissue healing process [23,40,41,42].

Growth factors (GFs) such as Transforming Growth Factor β (TGF-β), Fibroblast Growth Factor (FGF), and Platelet-Derived Growth Factor (PDGF) bind to their receptors and regulate downstream pathways including MAPK, PI3K/Akt, Wnt, and JAK-STAT, which control cell proliferation and differentiation and promote the formation of new tissue, thereby influencing various aspects of tissue repair. The extracellular matrix (ECM) plays a crucial role in the process of tissue regeneration, participating in the proliferation, migration, and differentiation of various cells. Composed of collagen, glycoproteins, proteases, and growth factors, the ECM forms an essential support structure and basis for cellular interactions during tissue regeneration and also serves as a medium between various cells and molecular mediators. Furthermore, tissue regeneration is also influenced by a combination of factors, including hemostasis, inflammation, oxidation, and bacterial infection. Overall, the natural product promotion of tissue healing encompasses a broad spectrum of actions.

**Figure 2:**
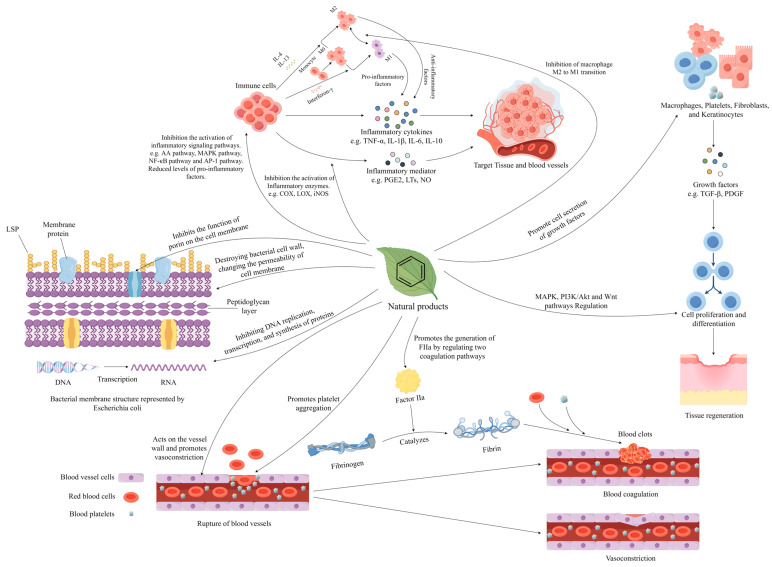
The complex pharmacological mechanisms of natural products, including anti-inflammatory, hemostatic, and antibacterial properties, and promoting tissue regeneration.

In summary, the main mechanism of natural products’ effects on hemorrhoids may be associated with promoting the coagulation process, suppressing inflammatory responses, inhibiting the growth of perianal pathogenic bacteria, and promoting the healing of tissue injuries [43,44,45,46], etc. Meanwhile, drawing upon the description of natural medicine effects presented in an Iranian review of hemorrhoid treatments [9], we hold the opinion that bioactive components from natural products considered to have theoretically potential hemorrhoid improvement capabilities if they possess all or at least two of these pharmacological actions. (The complex pharmacological mechanism of natural products is shown in Figure 2).

## 3. Pharmacological Effects of Bioactive Components from Natural Products

Existing animal experiments and clinical trials suggest that certain natural extracts or natural products exhibit substantial therapeutic effects on hemorrhoids. For instance, extracts from the fig leaf (*Ficus carica* L.) have demonstrated excellent hemorrhoid improvement ability in both animal studies and clinical trials. Animal experiments indicate that fig leaf extract significantly alleviates hemorrhoid-induced congestion, edema, inflammatory responses, and anal ulceration [47]; existing clinical trials show that the fig leaf extract healing rate exceeds 85% [48,49,50]. Interestingly, the efficacy of fig leaves on hemorrhoids even surpasses some commercially available topical hemorrhoid medications (*p* < 0.05) [51]. Another study using an animal hemorrhoid model suggests that extracts from *Cissus quadrangularis* may act as dual inhibitors of arachidonic acid metabolism, thus exhibiting potent anti-inflammatory properties. It also demonstrates the effects of venodilation, which is similar to those of flavonoids (a 90% diosmin and 10% hesperidin mixture) [52]. Additionally, studies have shown that extracts from *Capsella bursa-pastoris* (L.) Medik [53] and the tuber of *Amorphophallus paeoniifolius* (Dennst.) Nicolson [54] also possess hemorrhoid improvement effects due to their anti-inflammatory and antioxidant properties. Although these extracts have been confirmed to have hemorrhoid improvement effects, further research is needed to identify the specific bioactive components responsible for this ability.

Natural products such as flavonoids derived from certain plants and berries are documented in the American Society of Colon and Rectal Surgeons’ Clinical Practice Guidelines for the Management of Hemorrhoids as a class of effective ingredients for hemorrhoid improvement [8]. A clinical trial has also demonstrated that a mixture of flavonoids (diosmin, troxerutin, rutin, hesperidin, quercetin) is a safe and effective method for improving hemorrhoids, with minimal adverse reactions. The therapeutic effects are likely achieved through mechanisms such as hemostasis [55]. Ceylan Dönmez et al., through animal studies [56], have shown that extracts from eggplant (*Solanum melongena* L.) possess significant anti-inflammatory and hemorrhoid improvement activities, as they can reduce levels of the inflammatory cytokine TNF-α, decrease levels of vascular endothelial growth factor (VEGF), which is highly correlated with hemorrhoid [57], and reduce capillary permeability. Chlorogenic acid is hypothesized to be the primary bioactive component by their study.

Similarly, we believe that many natural products with hemorrhoid improvement potential exist in nature. They can broadly be categorized into polyphenols and flavonoids, terpenoids, polysaccharides, and other types, which are covered in this review.

### 3.1. Polyphenols and Flavonoids

Polyphenols are potential hemorrhoid improvement natural products with multiple therapeutic mechanisms, such as accelerating wound healing, preventing the progression of chronic wounds, suppressing inflammatory responses, and inhibiting the growth or virulent factors of *E. coli* without causing systemic side effects. For example, epigallocatechin gallate (EGCG) is considered the most abundant ingredient in green tea polyphenols and exhibits significant bioactivity. EGCG, in conjunction with mesenchymal stem cells (MSCs), promotes skin wound healing through a synergistic regulation of chronic inflammation [58,59,60]. Animal and cellular inflammation studies have shown that EGCG can significantly reduce the plasma concentrations of IL-1β, IL-6, IL-8, and TNF-α, as well as decrease levels of MDA [61]. Furthermore, in vitro studies have shown that EGCG can significantly reduce lipopolysaccharide (LPS)-induced CD80 expression and increase CD163 expression, demonstrating potential to reduce the inflammatory phenotype of macrophages. In vivo, EGCG primarily reduces inflammation by decreasing M1 macrophages and increasing regulatory T cells [62]. Additionally, as one of the most potent antimicrobial components in green tea, EGCG exhibits strong inhibitory effects on the T3SS (type III secretion system) virulent factor of enteropathogenic and enterohemorrhagic *E. coli* [63].

**Figure 3:**
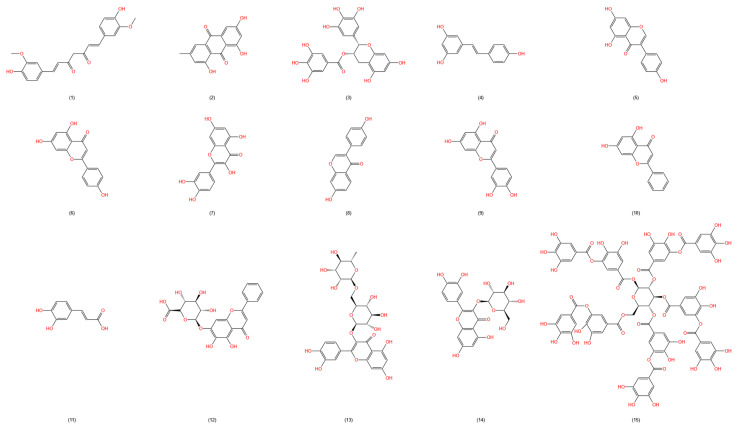
Chemical structures of polyphenols and flavonoids in the table. (1) Curcumin (CAS:458-37-7); (2) emodin (CAS:518-82-1); (3) epigallocatechin gallate (CAS:989-51-5); (4) resveratrol (CAS:501-36-0); (5) genistein (CAS:446-72-0); (6) apigenin (CAS:520-36-5); (7) quercetin (CAS:117-39-5); (8) daidzein (CAS:486-66-8); (9) luteolin (CAS:491-70-3); (10) chrysin (CAS:480-40-0); (11) caffeic acid (CAS:331-39-5); (12) baicalin (CAS:21967-41-9); (13) rutin (CAS:153-18-4); (14) isoquercitrin (CAS:21637-25-2); (15) tannic acid (CAS:1401-55-4).

Flavonoids are a class of low-molecular-weight polyphenols with multiple hydroxyl structures, which endow them with anti-inflammatory, antioxidant, and antimicrobial bioactivities, thus offering considerable hemorrhoid improvement potential [64], such as curcumin from turmeric (*Curcuma longa* L.), of the ginger family. Animal and cellular experiments have shown that curcumin can bind to TLRs and participate in regulating downstream inflammatory signaling pathways such as MAPK, NF-κB, and AP-1, significantly reducing levels of inflammatory mediators such as IL-1β, IL-6, TNF-α, NO, and iNOS, thus achieving anti-inflammatory effects [29,65]. Curcumin also inhibits the growth of *E. coli* by regulating various cell apoptosis pathways, such as reactive oxygen species (ROS) accumulation, membrane depolarization, calcium ion influx, and RecA protein expression [37]. The effects of curcumin in promoting tissue injury healing are related to its anti-inflammatory, anti-infection, and antioxidant activities, and are also associated with its involvement in tissue remodeling, granulation tissue formation, and collagen deposition [66]. Additionally, a review reported that curcumin inhibits the generation of activated coagulation factors X and thrombin, possessing strong antithrombotic capabilities [67]. Therefore, caution should be exercised when using it to improve severe hemorrhagic hemorrhoids. (A summary of polyphenols and flavonoids with hemorrhoid improvement potential is provided in Table 2, and chemical structures are shown in Figure 3).

**Table 2:** Polyphenols and flavonoids with potential effects on hemorrhoids and their pharmacological effects.

Bioactive Component	Natural Sources	Pharmacological Mechanism	Experimental Species	References
Curcumin	*Curcuma longa* L.	Inhibition of TNF-α, IL-1β, and IL-6 synthesis and NF-κB activation; promotes tissue repair by participating in tissue remodeling, granulation tissue formation, and collagen deposition processes; inhibits the growth of *E. coli*; inhibits the production of activated coagulation factor (FXa) and thrombin; has a strong antithrombotic ability.	RAW 264.7 cells/ Mice/*E. coli*/Review	[29,37,65,66,67]
Emodin	*Rheum officinale*	The levels of TNF-α, IL-β, and IL-6 in intestinal tissue were decreased, and COX-2 and mRNA of inflammation expression were inhibited; accelerated wound healing by promoting the synthesis of ECM and growth of granulation tissue; inhibition of *E. coli* energy metabolism.	Mice/RAW 264.7 cells/*E. coli*	[68]
Epigallocatechin gallate	Green tea	In coordination with MSC, it regulates chronic inflammation and promotes skin wound healing; inhibits the expression of CD80, promotes the expression of CD163, and significantly reduces the plasma concentrations of IL-1β, IL-6, IL-8, TNF-α, and MDA; inhibits the growth of *E. coli*.	Mice/Rats	[58,59,60,61,62]
Resveratrol	A number of plants	Downregulation of NF-Κb, MAPK, and AP-1 pathway activity; promotes the secretion of EGF, HGF, PDGF, and TGF-β1 growth factors by MSC.	Rats	[69,70,71]
Genistein	*Genista tinctoria* L.	Regulates the transforming TGF-β pathway; reduces the level of pro-inflammatory mediators and inhibits the activity of the NF-κB pathway; regulates gut microbiota composition.	Rats/Mice	[72]
Apigenin	*Apium graveolens* L and *Petroselinum crispum* (Mill.) Fuss	Inhibition of DNA gyrase activity inhibits *E. coli* growth by changing cell membrane permeability; inhibition of COX-2 and iNOS activity and IL-1β and IL-6 synthesis; inhibits the MAPK pathway; promotes platelet aggregation and coagulation pathways.	RAW264.7/*E. coli*/N/A	[73,74,75]
Quercetin	Onions, apples, broccoli, and other fruits and vegetables	Inhibits the production of TNF-α, IL-6, and IL-17 and promotes the synthesis of anti-inflammatory cytokine IL-10; effects on the transforming TGF-β pathway; interacts with platelets and promotes platelet coagulation and thrombus formation.	Rats/Review/N/A	[72,75,76]
Daidzein	*Glycine max* (Linn.) Merr	Inhibition of bacterial DNA topoisomerase activity and inhibition of bacterial nucleic acid expression; inhibiting the NF-kB pathway and the expression of COX-2 and iNOS; the levels of NO, IL-6, and TNF-α in cells were reduced.	*E. coli*/RAW 264.7 cells	[73,77]
Luteolin	*Reseda odorata* L.	Promotes the synthesis of anti-inflammatory cytokine IL-10 and inhibits the NF-κB and MAPK pathways; disruption of *E. coli* cell membrane integrity resulted in significant changes in cell morphology; acts on the exogenous coagulation pathway and the endogenous coagulation pathway to promote hemostasis.	Rats/*E. coli*/N/A	[75,78,79]
Chrysin	Not mentioned	Inhibition of PL-A2 expression and histamine release; promotes several stages of tissue repair.	Guinea pigs	[80]
Caffeic acid	Coffee beans	Local vasoconstriction; inhibition of fibrinolytic system; regulates the expression of genes involved in hemostasis and platelet activation; inhibited the activity of COX-2, reduced the synthesis of PGE2, and inhibited the synthesis of IL-8 and IL-1β.	Mice/Humans	[24,81,82]
Baicalin	*Scutellaria baicalensis* Georgi	By regulating IKK/IKB/NF-kB pathway, the levels of inflammatory mediators (IL-1β, TNF-α, PGE2, and MDA) in colon tissue were significantly decreased; has a broad-spectrum antibacterial effect.	Rats/Review	[36,83]
Rutin	*Ruta graveolens* L. and other plants	Antioxidant activity; reduces the levels of TNF-α, IL-6, COX-2, and IL-1β and inhibits the NF-κB pathway; promotes tissue healing by antioxidation and anti-inflammation.	Rats	[84]
Isoquercitrin	*Sophora japonica* L. and other plants	Reduces the expression of pro-inflammatory factors such as IL-6, IL-1β, and TNF-α; promotes the repair of skin injury, which may be related to the regulation of MAPK and JAK2-STAT3 signaling pathways; damages the cell membrane of *E. coli* and induces apoptosis of *E. coli.*	Mice/*E. coli*	[85]
Tannic acid	GALLA CHINENSIS and other plants	Inhibition of energy metabolism in *E. coli*; inhibits il-1β-induced expressions of IL-6, TNF-α, NO, and PGE2 in cells.	*E. coli*/Rats	[86,87]

### 3.2. Terpenoids

**Figure 4:**
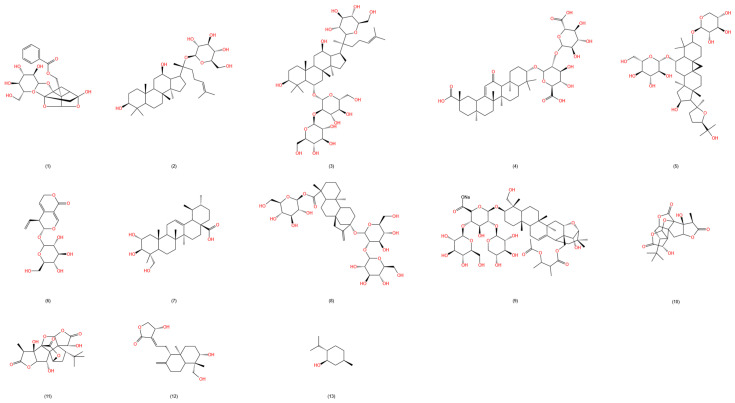
Chemical structures of terpenoids in the table. (1) Paeoniflorin (CAS:23180-57-6); (2) ginsenoside CK (CAS:39262-14-1); (3) ginsenoside RD (CAS:52705-93-8); (4) compound glycyrrhizin (CAS:1405-86-3); (5) astragaloside IV (CAS:84687-43-4); (6) gentiopicroside (CAS:20831-76-9); (7) Asiatic acid (CAS:464-92-6); (8) stevioside (CAS:57817-89-7); (9) sodium aescinate (CAS:20977-05-3); (10) ginkgolide A (CAS:15291-75-5); (11) ginkgolide B (CAS:15291-77-7); (12) andrographolide (CAS:5508-58-7); (13) menthol (CAS:89-78-1) (Total saponins of achyranthes bidens and soapnut saponin are a mixture, so there is no structural formula).

Terpenoids are defined by the general formula (C5H8)n and include oxygenated derivatives and variants with different degrees of unsaturation. They are widely distributed in nature and possess numerous bioactivities; we also considered them to have potential hemorrhoid improvement properties. For example, the compound glycyrrhizin derived from licorice (*Glycyrrhiza uralensis* Fisch.) can significantly reduce the concentrations of pro-inflammatory cytokines IL-1β and TNF-α in inflammatory animal models and also decrease the levels of inflammatory mediators such as PGE2 and NO by inhibiting the activity of COX-2 and iNOS [88]. Furthermore, studies have shown that compound glycyrrhizin hydrogel is a safe and effective wound healing agent, accelerating the repair phase of wounds through the modulation of macrophage responses in the inflammatory microenvironment [89]. It is noteworthy that the ability of compound glycyrrhizin to inhibit the growth of perianal pathogenic bacteria remains to be verified; however, current research indicates [90] that compound glycyrrhizin acts as a binder to the heat-labile enterotoxin of *E. coli*, inhibiting diarrhea caused by this toxin. It may ameliorate the increased abdominal pressure caused by diarrhea, thus potentially delaying the progression of hemorrhoids [5] (A summary of terpenoids with hemorrhoid improvement potential is provided in Table 3, and chemical structures are shown in Figure 4).

**Table 3:** Terpenoids with potential effects on hemorrhoids and their pharmacological effects.

Bioactive Component	Natural Sources	Pharmacological Mechanism	Experimental Species	References
Total saponins of achyranthes bidens	*Achyranthes bidentata* Bl.	Reduces the permeability of local capillaries; enhances the secretion of cortical hormones in the adrenal cortex and has obvious anti-inflammatory activity.	Rats	[91]
Soapnut Saponin	*Sapindus mukorossi* Saponin. and *Sapindus mukurossi* Gaertn	The infiltration of inflammatory cells in inflammatory tissues was inhibited, and the levels of TNF-α and IL-6 were significantly reduced; *E. coli* growth was inhibited at a concentration of 5 mg/mL.	Mice/*E. coli*	[92,93]
Paeoniflorin	*Paeonia lactiflora* Pall.	The levels of iNOS, TNF-α, and IL-1β were down-regulated, and the levels of IL-10 and TGF-β were up-regulated; regulates the conversion of M1 macrophages to M2 macrophages, thereby promoting wound healing.	Mice	[94]
Ginsenoside CK and Ginsenoside RD	*Panax ginseng* C. A. Mey.	Inhibits the production of PGE2 and the activation of COX-2; inhibition of *E. coli* energy metabolism.	Rats/mice/*E. coli*	[95,96]
Compound Glycyrrhizin	*Glycyrrhiza uralensis* Fisch.	The levels of IL-1β and TNF-α were significantly inhibited; inhibits the expression of COX-2 and iNOS and decreases the content of MDA, NO, and PGE2; promotes tissue repair by regulating macrophage responses in the inflammatory microenvironment; *E. coli* toxin inhibitors.	Mice/*E. coli*	[88,89,90]
Astragaloside IV	*Astragalus membranaceus* (Fisch.) Bunge	Inhibits the production of proinflammatory cytokines, inhibits the activation of NF-κB, and induces the production of anti-inflammatory cytokines; exhibits tissue repair capacity by promoting keratinocyte migration and promoting collagen synthesis.	Review/Rats	[97,98]
Gentiopicroside	*Gentiana lutea* L.	Promotes epithelial re-formation, granulation tissue growth, and collagen synthesis; the levels of MDA and IL-1β, TNF-α, IL-6, IL-17, and their related mRNA were significantly inhibited.	Rats	[99,100]
Asiatic Acid	*Centella asiatica* (L.) *Urb.*	Inhibits the release of pro-inflammatory factors (IL-17, IL-17F, IL-6, and TNF-α, etc.) in injured tissues, promotes the proliferation of fibroblasts, promotes the synthesis of EMC and collagen, and inhibits the growth of Staphylococcus aureus, *E. coli*, and other bacteria; promotes wound healing through multiple pathways.	Review	[101]
Stevioside	*Stevia rebaudiana*	Reduces the synthesis of TNF-α, IL-1β, and IL-6 and inhibits NF-κB in vitro, and inhibits NF-κB and MAPK pathway in vivo; in vitro, the growth of *E. coli* was significantly inhibited.	Mice/*E. coli*	[102]
Sodium Aescinate	*Aesculus chinensis Bunge*	Significantly decreased the level of TNF-α and increased the level of IL-10; it also significantly increased the activities of antioxidant enzymes superoxide dismutase (SOD), catalase (CAT), and glutathione peroxidase (GSH-Px); promotes tissue healing through anti-inflammatory and antioxidant effects.	Rats	[103]
Ginkgolide A	*Ginkgo biloba* L.	The activity of COX-2 was inhibited, the level of NO was reduced, and the expression of TNF-α, IL-6, and IL-1β was inhibited; effectively inhibits *E. coli* and other bacteria.	Mouse peritoneal macrophages/RAW264.7 cells/*E. coli*, etc./differentiated THP-1 cells	[104,105]
Ginkgolide B		The secretion levels of IL-1β, IL-6, and TNF-α were significantly decreased, the expression level of NO was decreased, and the activities of iNOS and COX-2 were inhibited; effectively inhibiting *E. coli* and other bacteria.	The murine microglial cell line BV2/*Staphylococcus aureus*/*E. coli*/*Klebsiella pneumonia*, etc.	[105,106]
Andrographolide	*Andrographis paniculata* (Burm.f.) Wall. ex Nees in Wallich	Inhibition of TRLs, such as inhibition of TLR3 or TLR4 agonist-induced NF-κB activation and COX-2 expression; in vitro antibacterial experiments showed that it could inhibit the growth of perianal pathogenic bacteria.	RAW264.7 cells/*Staphylococcus aureus*/*E. coli*/*Klebsiella pneumonia*, etc.	[107,108]
Menthol	*Mentha*	At different stages of wound healing, it suppresses the levels of TNF-α and IL-6 by reducing the mRNA expression of inflammatory factors and increases the expression of IL-10, reduces MPO activity, stimulates cell proliferation, and promotes granulation tissue formation; inhibits the formation of *E. coli* pili and reduces the virulence of *E. coli*.	Rats/*E. coli*, etc.	[109,110,111]

### 3.3. Polysaccharides

Polysaccharides, consisting of more than ten monosaccharides, are a class of macromolecular substances widely distributed in nature with numerous pharmacological effects. Therefore, we believe that there are certain polysaccharides that have potential hemorrhoid improvement capabilities. For instance, clinical trials have found that chitosan, derived from the exoskeleton of marine arthropods, can promote hemostasis by encouraging platelet aggregation and enhancing the aggregation of red blood cells, while also effectively absorbing exuded blood [112]. Chitosan contains a high concentration of basic amino groups, which carry a positive charge under slightly acidic pH conditions, enabling it to disrupt the cell walls of a range of bacteria, including *E. coli*, thus acting as an effective antibacterial agent [113,114]. Additionally, chitosan has been proven to regulate the probiotic populations on wound surfaces and stimulate the production of β-defensins, thereby promoting wound healing [112]. Based on these effects, chitosan can be considered for the improvement of various hemorrhagic hemorrhoids (A summary of polysaccharides with hemorrhoid improvement potential is provided in Table 4).

**Table 4:** Polysaccharides with potential effects on hemorrhoids and their pharmacological effects.

Bioactive Component	Natural Sources	Pharmacological Mechanism	Experimental Species	References
Bletilla striata polysaccharide	*Bletilla striata* (Thunb.) Reichb.f.	Promotes blood coagulation by promoting platelet aggregation and enhancing the function of coagulation system; increased secretion of TGF-β1 promotes tissue healing.	Review/Rats	[115,116]
Astragalus Polysacharin	*Scutellaria baicalensis* Georgi	The content levels of TNF-α and IL-1β were reduced; inhibits the growth of *E. coli* and Staphylococcus aureus.	Rats/*E. coli*/*Staphylococcus aureus*	[117,118]
Acanthopanax polysaccharide	*Eleutherococcus senticosus* (Rupr. & Maxim.) Maxim.	Decreases the levels of IL-1β and TNF-α; has a protective effect on intestinal mucosal injury by up-regulating the mRNA expression of epidermal growth factor and its receptor gene.	Mice	[119,120]
Chitosan	Biological crustacean	Inhibition of bacteria by charge action, metal chelation, gene binding; providing suitable environment for beneficial microorganisms to promote tissue healing; promoting platelet aggregation and promoting red blood cell aggregation; Anti-inflammatory properties.	Rats/Humans	[112,113]
Lycium barbarum polysaccharide	*Lycium chinense* Miller	Inhibition of *E. coli* growth; inhibition of M1-type macrophage differentiation and production of IL-1β and TNF-α	*E. coli*/RAW 264.7 cells	[118,121]
Momordica charantia polysaccharide	*Momordica charantia* L.	Inhibition of *E. coli* growth; inhibition of the NF-κB signaling pathway; it also up-regulates the level of IL-10 and decreases the levels of TNF-α, IL-1β, and IL-6.	*E. coli*/Mice	[122,123]
Ulvan Polysaccharides	*Ulva lactuca Linnaeus*, 1753	Inhibits the activity of COX-2, iNOS, and the MAPK pathway; it regulates the NF-κB pathway and inhibits the synthesis of interleukin, tumor necrosis factor, and NO; has hemostatic biological activity; changes the permeability of *E. coli* cell walls and inhibits the reproduction of *E. coli* by binding to DNA.	Review/*E. coli*	[124,125]
Lentinan	*Lentinula edode*	Inhibits the expression of TNF-α, IL-1β, and IL-6; regulate the composition of intestinal flora and improve intestinal health.	Weaned piglets	[126]
Ganoderma lucidum Polysaccharide	*Ganoderma lucidum* (Curtis) P. Karst.	Inhibits macrophage infiltration; decreases the expression of IL-1β, iNOS, and COX-2 by inhibiting the activation of the MAPK pathway; regulates the proportion and function of intestinal microbiota and protects the intestinal mucosa.	Mice/RAW 264.7 cells	[127]
Fucoidan, FPS	Brown algae and echinoderms	Stimulates the production of GFs, promotes cell proliferation and differentiation, and enhances collagen synthesis; inhibits the production of inflammatory factors and inhibits the activation of pro-inflammatory signaling pathways; has a heparin-like effect; intravenous administration of 5 mg/kg in mice had an antithrombotic effect without increasing clotting time (may have effects on thrombotic hemorrhoids).	Humans/Mice	[128,129]

### 3.4. Other Types

**Figure 5:**
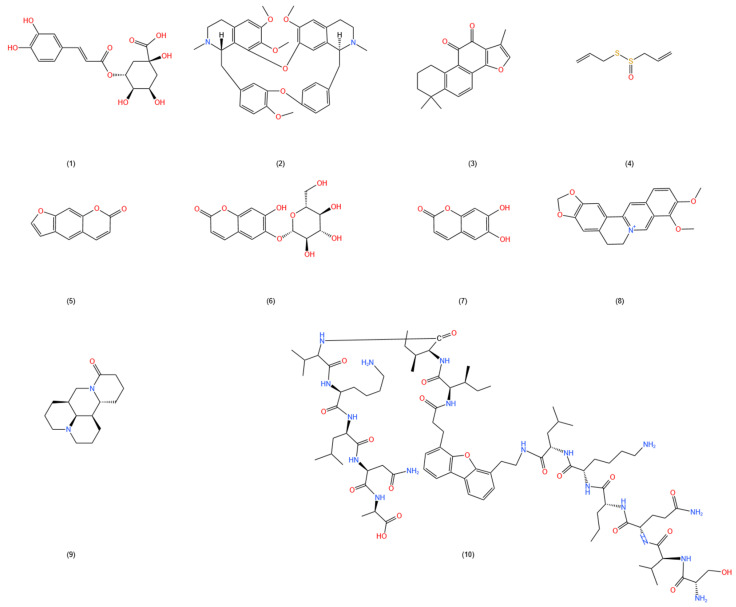
Chemical structures of other types in the table. (1) Chlorogenic acid (CAS:327-97-9); (2) tetrandrine (CAS:518-34-3); (3) tanshinone IIA (CAS:568-72-9); (4) allicin (CAS:539-86-6); (5) psoralen (CAS:66-97-7); (6) esculin (CAS:531-75-9); (7) esculetin (CAS:305-01-1); (8) berberine (CAS:2086-83-1); (9) matrine (CAS:519-02-8); (10) galectin-1 (CID:169450756) (has no CAS number).

In addition to the types of natural products mentioned above, many other types such as peptides and proteins, phenylpropanoids, coumarins, and alkaloids also possess numerous bioactivities, which possess potential hemorrhoid improvement capabilities. For example, peptides and proteins isolated from animal sources have been studied using mouse and skin wound models, as well as isolated and cultured fibroblasts. Research has shown that galectin-1 can promote tissue healing by regulating the generation of myofibroblasts and inducing myofibroblast activation through the NRP1 binding and the Smad3/NOX4 pathway [130]. Galectin-1 can also enhance the synthesis of the ECM via the PI3K/Akt pathway [131]. Additionally, in a mouse model of colitis, galectin-1 demonstrated the ability to reduce levels of pro-inflammatory cytokines in plasma and mucosal tissue [132]. Certain plant alkaloid extracts can regulate inflammation and promote tissue repair. Existing experiments indicate that tetrandrine from dried root of *Stephania tetrandra* S. Moore significantly elevates the cAMP (a cellular second messenger) concentration in inflammatory leukocytes and inhibits phosphodiesterase (PDE) activity, thereby exhibiting anti-inflammatory activity [133]. Tetrandrine also promotes tissue repair by regulating the PI3K/AKT signaling pathway, inhibiting apoptosis, and reducing inflammatory responses [134]. (A summary of other types with hemorrhoid improvement potential is provided in Table 5, and chemical structures are shown in Figure 5).

**Table 5:** Other types (including peptides and proteins, phenylpropionic acids, alkaloids, phenanthraquinones, organic sulfur compound, and coumarins) with potential effects on hemorrhoids and their pharmacological effects.

Bioactive Component	Categories	Natural Sources	Pharmacological Mechanism	Experimental Species	References
Galectin-1	Peptides and proteins	Vertebrate animals	Regulation of the Smad3/NOX4 signaling pathway in myofibroblasts induces myofibroblast activation, migration, and proliferation; promotes ECM synthesis through the PI3K/Akt pathway; reduces proinflammatory cytokine levels in plasma and mucosal tissues.	Rats	[130,131,132]
Chlorogenic acid	Phenylpropionic acids	*Solanum melongena* L.	Significantly reduces the level of TNF-α, VEGF, and capillary permeability in the mucosa; may have hemorrhoid improvement effect.	Rats	[56]
Tetrandrine	Alkaloids	*Stephania tetrandra* S. Moore	Increases cAMP concentration in inflammatory cells and inhibits PL-A2 activation; regulation of the PI3K/AKT signaling pathway promotes tissue generation, by inhibiting apoptosis, and reduces inflammatory response.	Rats	[133,134]
Tanshinone IIA	phenanthraquinones	*Salvia miltiorrhiza* Bunge	Significantly inhibits the levels of TNF-α, IL-1β, and IL-6 and inhibits the activation of NF-κB; inhibits platelet activation and thrombosis by regulating Akt/ERK and cSrc/RhoA signaling pathways (may have effects on thrombotic hemorrhoids.)	Humans/Mice	[135,136]
Allicin	organic sulfur compound	*Allium sativum* L.	Inhibits the expression of P38 and JNK inflammatory pathways and NF-κB nuclear factor; through multiple inhibitory effects, it inhibits sulfhydryl-dependent enzyme systems of various perianal pathogens including *E. coli*.	Rats/*E. coli*, etc.	[137,138]
Psoralen	Coumarins	*Ficus carica* L. and *Psoralea corylifolia* Linn.	Hemostatic effect; inhibits the TLR4/NF-κB signaling pathway, thereby inhibiting the expression of TNF-α and ILs.	Review/ Human periodontal ligament cells	[24,139]
Esculin	Coumarins	*Fraxinus chinensis* Roxb.	The activity of the TLR/NF-κB pathway was inhibited, and the expression of TNF-α, ILs, COX-2, iNOS and other inflammatory mediators was reduced; the MIC was 20 mg·mL^−1^ for Staphylococcus aureus and 10 mg·mL^−1^ for *E. coli*.	Mice/*Staphylococcus aureus*/*E. coli*	[140,141]
Esculetin	Coumarins		Inhibition of the expression of *E. coli* curli genes and motility genes reduces the production of pili, and inhibition of the expression of the Shiga-like toxin gene stx2 reduces the virulence of *E. coli*; inhibits the production of proinflammatory cytokines, inflammatory mediators, and inhibits the NF-κB pathway.	*E. coli*/RAW264.7 cells/Rats	[142]
Berberine	Alkaloids	*Coptis chinensis* Franch.	Inhibits the effect of *E. coli* toxin and can inhibit Staphylococcus aureus in vitro; reduces intestinal injury induced by LPS by inhibiting the NF-κB and MAPK pathways and inhibiting COX-2 activity.	*E. coli*/*Staphylococcus aureus*/Review	[143,144]
Matrine	Alkaloids	*Sophora flavescens* Aiton	By inhibiting the NF-κB pathway, it inhibits the synthesis of pro-inflammatory factors TNF-α and IL-1β and inhibits the expression of COX-2 and iNOS, thereby reducing capillary vascular permeability and pain; inhibits the growth of bacteria such as Staphylococcus aureus and *E. coli*; the inhibitory mechanism may be the inhibition of biofilm formation.	Mice/*Staphylococcus aureus*/*E. coli*	[145]

## 4. Clinical Trials

In recent decades, clinical trials on hemorrhoids have largely focused on compound preparations and natural extracts, yet there is a conspicuous lack of clinical trials concerning the treatment of hemorrhoids with bioactive components from natural products. This has resulted in a substantial research gap in this field. According to our literature review, clinical trials primarily assess the therapeutic effects of compound preparations and natural extracts on hemorrhoids, with most studies concentrating on superficial observations such as changes in hemorrhoid size and symptom improvement [44,45,46,48,49,50,146,147], while in-depth studies on pharmacological effects are notably lacking. Furthermore, we have identified several issues with existing clinical trials: there is a lack of investigation into the optimal dosage and toxicological aspects of clinical medications; some clinical trial designs lack scientific rigor, such as not taking into account the individual characteristics of different patients; and the sample sizes in clinical trials are generally small. Therefore, before natural products can be standardized as treatments for hemorrhoids, more clinical research in the future is needed using more rigorous, systematic, and scientific methods, as well as higher-quality samples. We also hope that future research will conduct more targeted pharmacological studies and clinical trials on the treatment of hemorrhoids with natural products, to demonstrate their safety and efficacy.

## 5. Conclusions

In summary, products found in nature have potential for hemorrhoid improvement through mechanisms such as promoting the coagulation process, inhibiting inflammatory responses, preventing the proliferation of perianal pathogenic bacteria, and promoting the repair of tissue damage. Currently, except for a few flavonoids, there are only a few studies on the use of bioactive components from natural products for hemorrhoid improvement, with even fewer studies conducted at the cellular and molecular levels. Therefore, we have searched for several bioactive components with potential to improve hemorrhoids. 

Using natural products with well-defined pharmacological mechanisms to improve hemorrhoids is a cost-effective and environmentally friendly approach. However, we must admit that most existing research derives from animal or cellular models, and their efficacy in humans has not been fully validated. Moreover, studies on the optimal dosage of these natural products, potential medication interactions, used alone or in combination, and their mechanisms against hemorrhoids are also lacking sufficient experimental support. Despite these limitations, the findings still hold some reference value. 

Natural products such as polyphenols, flavonoids, terpenes, polysaccharides, coumarins, and even peptides and proteins isolated from plants, animals, fungi, and algae are potential hemorrhoid drugs, prevention products, and medication add-on ingredients. After review, we believe that components like chlorogenic acid, epigallocatechin gallate, curcumin, quercetin, isoquercetin, luteolin, apigenin, compound glycyrrhizin, Asiatic acid, menthol, and chitosan show substantial potential hemorrhoid improvement effects. Interestingly, most of the above bioactive components can be obtained from some foods such as eggplant (chlorogenic acid); green tea (epigallocatechin gallate); turmeric (curcumin); onions, apples, and broccoli, etc. (quercetin); okra (isoquercetin) [148]; celery (apigenin); and mint (menthol). Therefore, the intake of these foods in the diet may prevent hemorrhoids or alleviate hemorrhoid symptoms to some extent. Additionally, those bioactive components could also provide new options for the development of new hemorrhoid products. 

In the future, except for increasing the number of studies in this area, we also recommend that research into the pharmacological mechanisms of natural products against hemorrhoids should start at the cellular and molecular levels, which could provide more scientific references for the development of new hemorrhoid products. Additionally, studying the cellular and molecular mechanisms of natural products in improving hemorrhoid symptoms not only complements existing research methods but could also represent a potential future research direction.

At the same time, future research needs to be more scientific and systematic, involving representative animal experiments and clinical trials. Research should also be conducted to explore the therapeutic mechanisms of hemorrhoids, use-pattern, optimal dosage, and medication interactions of these bioactive components. Furthermore, whether components structurally similar to these also possess similar biological activities remains to be further explored.

Overall, the variety of potential hemorrhoid improvement natural products is vast, and the resources are abundant, suitable for reasonable development and utilization as hemorrhoid products. Through this review, we hope to provide a scientific reference for the search and development of new hemorrhoid medications and prevention products, as well as providing reference for existing hemorrhoid medications composition improvement and offering a possible novel avenue for future research into the pharmacological mechanisms of hemorrhoid products. Furthermore, we also summarize some existing problems waiting for further exploration in the future.

## Figures and Tables

**Table 1:** Goligher classification system: classification criteria and symptoms of internal hemorrhoids.

Degree I	Accompanied by hematochezia, dripping blood. After defecation, the bleeding stopped. The hemorrhoid did not come out of the anus.
Degree II	It is often accompanied by hematochezia and prolapse of the hemorrhoid from the anus, which can recover spontaneously after defecation.
Degree III	Occasionally accompanied by hematochezia. Defecation, standing for a long time, weight-bearing, and a series of behaviors that increase intra-abdominal pressure can make the hemorrhoid come out and need to be restored with hand assistance.
Degree IV	Occasionally accompanied by hematochezia. The hemorrhoid cannot recover or automatic prolapse after the recovery. It may be accompanied by mucosal ulceration in the anal dentate line area, infection, vascular exposure, and severe pain.

## Data Availability

Not applicable.
